# Coach–Athlete Perceptual Alignment in Youth Soccer: A Descriptive Multi-Case Study of Leadership Perceptions and Match-Observed Behaviors

**DOI:** 10.3390/bs16091586

**Published:** 2026-09-07

**Authors:** Ionut-Alexandru Buda, Alexandra Mihaela Stănilă, Silvia Popescu, Ralph-Alexandru Erdelyi, Bogdan Almajan

**Affiliations:** 1 Faculty of Physical Education and Sport, West University of Timisoara, Vasile Parvan Boulevard, No. 4, 300223 Timisoara, Romania; ionut.buda@e-uvt.ro (I.-A.B.); alexandra.rusu@e-uvt.ro (A.M.S.); silvia.popescu@e-uvt.ro (S.P.); bogdan.almajan@e-uvt.ro (B.A.); 2 Faculty of Electronics, Telecommunications and Information Technologies, Polytechnic University of Timisoara, 1 Mihai Viteazu Ave., 300222 Timisoara, Romania

**Keywords:** coach–athlete perceptual alignment, coaching leadership, Multidimensional Scale of Leadership in Sport, Coach Analysis and Intervention System, youth soccer, behavioral observation

## Abstract

Coaching leadership is experienced through both subjective perceptions and observable actions, but these sources do not represent equivalent constructs. This descriptive multi-case study examined coach–athlete perceptual alignment and match-observed behaviors in four elite youth soccer coach–team cases. Four male youth soccer coaches and 64 male athletes (14–18 years) from a single Romanian elite soccer academy participated in the study. Athletes completed the Multidimensional Scale of Leadership in Sport (MSLS) before and after an official match; each coach completed one post-match self-rating referring to that match. Each coach was compared only with his corresponding team’s post-match mean. Dimension-specific discrepancy scores and a mean absolute discrepancy (MAD) index were calculated. Pre–post comparisons used Wilcoxon signed-rank tests with Holm correction. One match per coach was coded descriptively using the Coach Analysis and Intervention System (CAIS). The MAD index was lowest for the U17 case (1.93) and highest for the U18 case (4.35). After Holm correction, post-match scores were lower for Inspiration, Individualization, and Support. Instruction was highly rated in the MSLS and was the most frequent CAIS category in all four matches (25.2–41.6%), representing a parallel descriptive pattern rather than formal agreement. The findings are specific to four coach–team cases and provide complementary, preliminary perspectives on perceived and match-observed leadership. The single-match, single-observer design and absence of observational reliability assessment preclude characterization of habitual coaching behavior.

## 1. Introduction

Youth soccer represents a complex developmental environment in which performance demands, educational processes, psychosocial adaptation, and interpersonal relationships continuously interact (Alfermann et al., 2005; Black & Weiss, 1992). Within this context, coaches occupy a central role, influencing not only athletes’ technical and tactical preparation but also their motivational climate, emotional regulation, communication experiences, and overall well-being (Côté & Gilbert, 2009; Lyle, 2005). This role becomes particularly important in elite soccer academies, where competitive pressure and performance expectations emerge early and may substantially shape athletes’ sporting experiences, psychological functioning, and long-term engagement in sport (Anshel et al., 1986; Simon & Martens, 1979; Wong et al., 1993; C. Cushion et al., 2012).

Over the last two decades, increasing attention has been directed toward leadership processes in sport and the way coaching behaviors influence athletes’ experiences and developmental outcomes (Gomes et al., 2021; Jin et al., 2022). Contemporary models of coaching effectiveness no longer define successful coaching exclusively through competitive performance. Instead, effective leadership has increasingly been associated with the ability to integrate tactical instruction, emotional support, interpersonal adaptability, and athlete-centered communication within a coherent behavioral framework (Côté & Gilbert, 2009; Lyle, 2005). In this regard, transformational leadership approaches have gained considerable attention in sport psychology, emphasizing behaviors such as inspiration, individualized consideration, positive reinforcement, and the development of meaningful coach–athlete relationships (Gomes, 2014; Gomes & Resende, 2014).

The relationship between coaching leadership and athlete motivation has also been extensively examined from the perspective of Self-Determination Theory (SDT; Deci & Ryan, 2000). According to this framework, autonomy-supportive coaching environments are associated with greater athlete motivation, satisfaction, persistence, emotional well-being, and long-term engagement in sport (Coatsworth & Conroy, 2006; Conroy & Coatsworth, 2004). Coaches who encourage participation, provide constructive feedback, and facilitate supportive communication may positively influence athletes’ intrinsic motivation and perceived competence (Black & Weiss, 1992; Soyer et al., 2014). Conversely, authoritarian, punitive, or emotionally distant coaching styles may negatively affect athletes’ emotional experiences, confidence, and psychosocial adaptation within competitive sport environments (Jin et al., 2022; Potrac et al., 2002). These findings suggest that athletes’ perceptions of coaching behaviors may be just as important as the behaviors themselves, as athletes ultimately respond to how leadership is experienced rather than how it is intended by the coach.

Despite the recognized importance of coaching leadership, coaches may evaluate their own behavior differently from their athletes, particularly regarding support, feedback, and management (Alfermann et al., 2005; Bortoli et al., 1995; Gardner et al., 1996; Westre & Weiss, 1991). In this study, perceptual alignment refers specifically to the numerical proximity between each coach’s post-match MSLS self-rating and the mean post-match rating of athletes from the same team. Alignment was treated as a continuous descriptive property: smaller dimension-specific discrepancies indicated closer alignment, whereas larger absolute discrepancies indicated greater perceptual difference. It was not treated as categorical or as formal statistical agreement.

Research investigating coaching leadership has generally followed two methodological directions. The first involves questionnaire-based approaches designed to assess perceptions of leadership and interpersonal functioning (Gomes & Resende, 2014; Gomes et al., 2021), whereas the second relies on systematic observational methods capable of quantifying coaching behaviors during training sessions or competition (C. J. Cushion et al., 2012; Lacy & Darst, 1985). Each perspective provides distinct yet complementary information. Perceptual instruments capture the subjective experiences and interpretations of athletes and coaches, whereas observational systems quantify overt coaching behaviors in ecological sporting environments (C. J. Cushion et al., 2012; Lyle, 2005).

One of the most widely used observational frameworks in coaching research is the Coach Analysis and Intervention System (CAIS), which categorizes verbal and non-verbal coaching behaviors during sport participation (C. J. Cushion et al., 2012). Through this system, behaviors such as instruction, praise, questioning, corrective feedback, silence, scolding, and interaction management can be systematically quantified during competition. Previous observational studies in soccer have consistently shown that instructional behaviors constitute a prominent component of coaching activity during both training and official matches, reflecting the traditionally directive structure of soccer coaching environments (C. J. Cushion & Jones, 2001; C. Cushion et al., 2012; Ford et al., 2010; Partington & Cushion, 2013; Smith & Cushion, 2006).

At the same time, multidimensional leadership models have become increasingly relevant for understanding coaching effectiveness. The Multidimensional Scale of Leadership in Sport (MSLS) evaluates transformational, transactional, and management-related leadership dimensions and provides a broader perspective on interpersonal coaching dynamics (Gomes & Resende, 2014; Gomes et al., 2021). However, despite the growing literature on coaching leadership, to our knowledge, few studies have simultaneously examined coaches’ self-perceptions, athletes’ perceptions, and systematically observed coaching behaviors during official competition in elite youth soccer. Consequently, it remains unclear whether coaches, athletes, and systematic observations portray a similar picture of leadership behaviors during competition.

Importantly, the MSLS and CAIS assess related but non-equivalent aspects of coaching. The MSLS captures broad subjective leadership perceptions (Gomes et al., 2021), whereas CAIS records frequencies of discrete verbal and non-verbal behaviors during a selected observation period (C. J. Cushion et al., 2012). Their integration was therefore used only to identify cautious parallel, partially parallel, or contrasting descriptive patterns. CAIS frequencies were not treated as behavioral equivalents of MSLS dimensions or as validation of questionnaire scores.

Therefore, the aim of this descriptive multi-case study was to examine coach–athlete perceptual alignment within four elite youth soccer coach–team cases and to complement these case-specific MSLS findings with preliminary descriptions of behaviors observed during one official match per coach. It was expected that coach self-ratings would exceed corresponding athlete ratings for selected relational and management dimensions and that Instruction would be frequently observed during the selected matches. No age-related or developmental hypotheses were tested because each category was represented by one coach.

## 2. Materials and Methods

### 2.1. Study Design

The study used a descriptive convergent multi-case design involving four coach–team cases. The core unit for the observational component was the coach–team case, not the individual athlete. MSLS and CAIS data were analyzed independently and integrated only at the descriptive interpretation stage.

The MSLS assessed coach self-perception and athlete perception across nine leadership dimensions. CAIS documented discrete behaviors observed during one selected official match per coach. Because these sources differ in construct, temporal scope, and unit of measurement, integration was intended to provide complementary contextual information rather than formal agreement or validation.

Each age category was represented by one coach; consequently, the four cases could not distinguish age-category effects from coach-, team-, or match-specific influences. All between-case comparisons were descriptive, and no population-level or developmental inference was intended.

### 2.2. Study Setting

The study was conducted within a single Romanian elite youth soccer academy competing at national level. The academy included structured developmental teams across the U15, U16, U17, and U18 competitive categories and operated within a performance-oriented training environment focused on long-term athlete development and national competition.

Data collection occurred during the regular junior league season under naturalistic conditions. All four observed matches were played at home against opponents from the corresponding age category and ended in victories. No injuries, dismissals, or other unusual events were reported. Exact scorelines, score progression, opponent strength, substitutions, and detailed performance indicators were unavailable for retrospective analysis.

The use of a single-academy setting allowed the perceptual and observational components of the study to be examined within a relatively consistent organizational and competitive environment. At the same time, the findings reflect the characteristics of this specific academy and should not be considered representative of youth soccer academies more broadly.

Because each competitive category was led by a different coach, the four participating coach–team units were treated as distinct cases embedded within the same institutional setting.

### 2.3. Participants

The study included four male head coaches, aged 30–50 years, and 64 male athletes aged 14–18 years. Each coach had worked with his respective team for approximately two years. The under-15 (U15), under-16 (U16), and under-18 (U18) coaches held Union of European Football Associations (UEFA) A licenses, whereas the under-17 (U17) coach held a UEFA B license. Total career coaching experience was not formally recorded.

Athletes were distributed as follows: U15 (n = 18), U16 (n = 17), U17 (n = 15), and U18 (n = 14). Eligibility required official academy registration, regular participation in training and league matches, and at least six months under the respective coach. Playing position and starter/substitute status were not used as exclusion criteria.

Participation was voluntary. Written informed consent was obtained from all coaches and athletes prior to data collection. For participants younger than 18 years of age, written informed consent was additionally obtained from their parents or legal guardians.

### 2.4. Assessment of Leadership Perceptions

Leadership perceptions were assessed using the Multidimensional Scale of Leadership in Sport (MSLS) developed by Gomes et al. (2021). The MSLS is a multidimensional self-report instrument designed to assess transformational, transactional, and management-related leadership behaviors within sport settings (Gomes, 2014; Gomes & Resende, 2014) and has demonstrated satisfactory psychometric properties in previous research (Gomes et al., 2021).

The instrument comprises 36 items organized into nine leadership dimensions: Vision, Inspiration, Instruction, Individualization, Support, Positive Feedback, Negative Feedback, Active Management, and Passive Management. Each dimension is represented by four items.

Parallel MSLS versions were used. Athletes completed the observer version approximately 30 min before kick-off and within 30 min after the match. Each coach completed the self-report version once, within 30 min after the match, and evaluated his behavior during the match just played. Accordingly, coach–athlete alignment analyses used only the corresponding post-match athlete ratings.

The questionnaire was administered in Romanian. Documentation of a formal Romanian validation or a translation/back-translation procedure was unavailable. Internal consistency was therefore evaluated for the present sample but was not interpreted as evidence of linguistic, cultural, or construct validity.

All items were scored using a five-point Likert scale, ranging from 1 (strongly disagree) to 5 (strongly agree). Scores for each leadership dimension were calculated by summing the corresponding item responses, with higher scores indicating stronger perceptions of the respective leadership behavior.

The internal consistency of all MSLS dimensions was evaluated using Cronbach’s alpha (α) and McDonald’s omega (ω) coefficients. Complete psychometric results for all subscales, including those demonstrating comparatively lower reliability, are reported in Section 3 to provide a comprehensive evaluation of the instrument’s performance within the present sample.

The MSLS constituted the perceptual component. Coach and athlete scores were compared within the same case and assessment context; MSLS findings were then considered alongside CAIS observations only through a predefined descriptive integration matrix.

### 2.5. Systematic Behavioral Observation

Behaviors displayed during the selected official matches were examined using the Coach Analysis and Intervention System (CAIS), developed and validated by C. J. Cushion et al. (2012). The system classifies observable verbal and non-verbal coaching actions occurring during sport participation.

The recordings were imported into the CAIS software (https://www.isportsanalysis.com/coach-analysis-intervetion-system.php, accessed on 1 September 2026). The first author reviewed each match offline and selected the applicable CAIS behavioral category whenever an event occurred. Absolute event counts were recorded for each category and expressed as percentages of all coded behaviors within the corresponding coach–team case. The resulting categories provided a descriptive account of behaviors during official match play and supported cautious conceptual comparison with the MSLS dimensions.

One complete official league match was available for each coach–team case. The U15 match had a regulation duration of 80 min (2 × 40 min), whereas the U16, U17, and U18 matches had regulation durations of 90 min (2 × 45 min). Each available recording was coded in full. Exact stoppage time was not recorded separately and could not be reported retrospectively. Because CAIS findings were expressed as proportions of total coded events rather than event rates per minute, differences in match duration should be considered when comparing cases. These observations captured only the selected matches and were not intended to characterize stable or habitual coaching behavior across a season.

CAIS coding was completed by the first author before the MSLS results were examined; the observer was therefore blinded to the questionnaire findings during behavioral classification. MSLS and CAIS results were integrated only after their separate analyses.

Coding was performed by a single observer. Neither inter-rater nor intra-rater reliability was assessed, and retrospective reliability testing was not possible. Consequently, the CAIS findings are presented exclusively as preliminary descriptive observations. No inference regarding coding reliability, habitual coaching patterns, instructional quality, or behavioral effectiveness is made. The match recordings were retained by the club. Access to the present analysis was restricted to the first author, and raw recordings were not publicly disseminated.

### 2.6. Integration of Quantitative and Observational Data

The MSLS and CAIS datasets were analyzed independently and subsequently integrated through a transparent exploratory matrix. Integration was limited to conceptually related dimensions and categories, with the rationale and limitations of each comparison stated explicitly.

Instruction was considered alongside CAIS Instruction; Positive Feedback alongside Praise and general/specific positive feedback; and Negative Feedback alongside Scold and Specific Negative Feedback. Support and the management dimensions had no direct CAIS equivalents and were considered only contextually. The matrix classified cross-method findings cautiously as parallel, partially parallel, contrasting, or not directly comparable. Frequency of an observed action was not interpreted as the quality, effectiveness, or psychological meaning of that action.

No inferential correlations or agreement coefficients were calculated between MSLS and CAIS because only four coach–team cases were available and the constructs were non-equivalent.

### 2.7. Statistical Analysis

Statistical analyses were performed using JASP (Version 0.19; JASP Team, Amsterdam, The Netherlands). Data organization, verification, and graphical presentation were conducted using Microsoft Excel and reproducible Python (Version 3.14; Python Software Foundation, Wilmington, DE, USA) scripts.

Descriptive statistics included means, standard deviations, medians, interquartile ranges, frequencies, and percentages. Internal consistency of each MSLS dimension was evaluated using Cronbach’s alpha (α) and McDonald’s omega (ω). These coefficients describe score consistency in the present sample and do not establish validation of the Romanian-language version. Paired pre–post athlete scores were compared using Wilcoxon signed-rank tests. Because athletes were nested within four teams and shared the same coach and match context, pooled tests were interpreted as exploratory individual-level comparisons rather than independent population-level evidence. Case-wise pre–post medians were additionally reported descriptively.

To facilitate interpretation of the magnitude of the observed differences, effect sizes (r) were calculated for the Wilcoxon signed-rank tests using the formula:

$$r=\frac{Z}{\sqrt{N}}$$

Effect sizes were interpreted according to conventional thresholds as small (r ≥ 0.10), medium (r ≥ 0.30), and large (r ≥ 0.50).

Holm correction was applied across the nine MSLS pre–post comparisons; both nominal and adjusted *p*-values are reported, and statistical significance is interpreted using adjusted *p*-values.

Coach–athlete perceptual alignment was analyzed within each case using the coach’s single post-match score and the corresponding team’s post-match mean. For each MSLS dimension, the signed discrepancy was defined as D = coach score-team mean. Positive values indicate higher coach self-ratings, negative values indicate higher athlete ratings, and values closer to zero indicate closer descriptive alignment.

For each case, a mean absolute discrepancy (MAD) index was calculated as the mean of |D| across the nine MSLS dimensions. No inferential congruence or agreement statistic was applied because only four coaches were available, one per team, with unequal athlete clusters; an ICC or comparable coefficient would be unstable and would imply unsupported inferential precision. Team-level MSLS-CAIS Spearman correlations were removed. Integration across methods was descriptive and based on the predefined conceptual matrix rather than inferential association testing.

All analyses were intended to characterize the four coach–team cases investigated. They do not support age-category effects, stable coach profiles, causal attribution to match context, or broad generalization.

### 2.8. Ethical Considerations

The study was conducted in accordance with the principles of the Declaration of Helsinki regarding research involving human participants.

Participation was voluntary. Coaches and athletes received written and verbal study information. Written consent was obtained from participating coaches and athletes; for minors, parents or legal guardians additionally provided written consent. Match recording was routinely conducted by the club under parental authorization. The study involved non-invasive questionnaires and secondary behavioral coding of routinely recorded official matches. The recordings focused on the head coach and were analyzed without altering match or training procedures.

Questionnaire data were anonymized before analysis. Video files remained under club control, access was restricted, and raw recordings were not shared because they contain identifiable minors and team environments.

## 3. Results

### 3.1. Sample Characteristics

The study included four male youth soccer coaches and 64 male athletes from a Romanian elite soccer academy competing at national level. Athletes were distributed across four competitive teams corresponding to the U15 (n = 18), U16 (n = 17), U17 (n = 15), and U18 (n = 14) categories.

The four coach–team cases and their match context are summarized in Table 1. All teams completed both athlete assessment time points. Coach self-ratings were collected once after the match. The demographic and competitive characteristics of the study participants are summarized in Table 1.

### 3.2. Psychometric Properties of the Multidimensional Scale of Leadership in Sport

The internal consistency of the MSLS was evaluated using both Cronbach’s alpha (α) and McDonald’s omega (ω) coefficients (Table 2).

Cronbach’s alpha values ranged from 0.649 to 0.926, and McDonald’s omega coefficients ranged from 0.689 to 0.926. Eight of the nine subscales demonstrated acceptable-to-excellent internal consistency in this sample.

Passive Management showed lower internal consistency (α = 0.649; ω = 0.689) and should be interpreted cautiously. These estimates do not establish validity of the Romani-an-language administration.

The reliability analysis supported use of the subscale scores for exploratory description in the present sample, while linguistic and cultural validity remain unverified.

### 3.3. Athletes’ Leadership Perceptions Across Coach–Team Cases

Table 3 presents descriptive MSLS scores combined across the pre- and post-match athlete assessments. These combined values provide an overall case description and are not used for coach–athlete alignment, which is based exclusively on post-match ratings.

Across the combined assessments, Instruction was generally among the highest-rated dimensions and Passive Management among the lowest. Differences between U15, U16, U17, and U18 describe individual coach–team cases and do not represent age-category effects.

**Figure 1:**
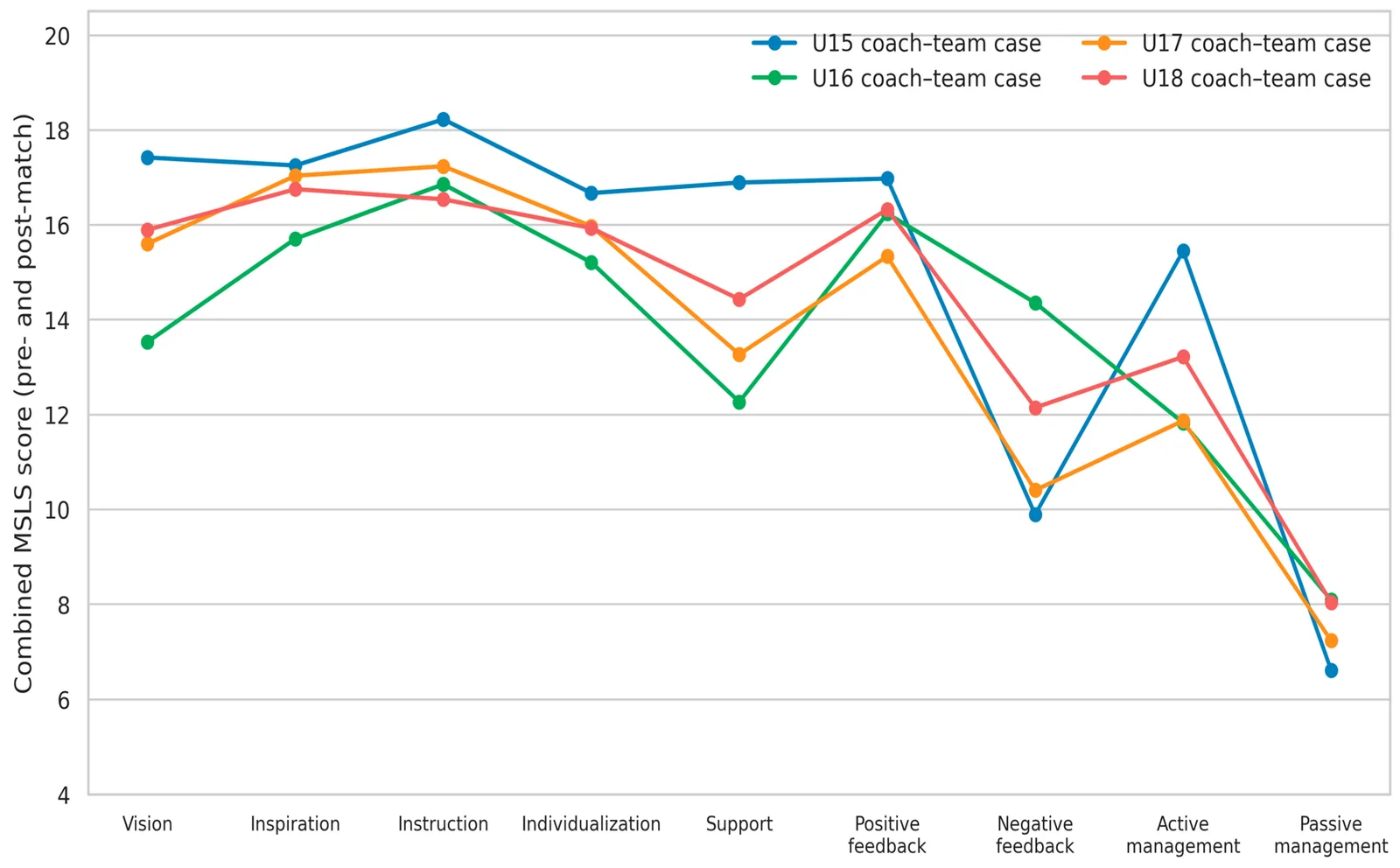
Combined pre- and post-match athlete MSLS scores across the four coach–team cases. Lines describe the four individual cases and should not be interpreted as age-related patterns.

Figure 1 provides a simplified visualization of these combined descriptive patterns.

To complement the descriptive statistics, Figure 1 illustrates the overall leadership perception profiles across the four coach–team cases investigated. The radar plot facilitates the visual comparison of the nine MSLS leadership dimensions while remaining descriptive in nature.

### 3.4. Coach–Athlete Perceptual Congruence

Each coach’s single post-match MSLS self-rating was compared exclusively with the post-match mean of athletes from the corresponding team. Table 4 reports coach scores, team means, signed discrepancies, and the case-level MAD index.

The MAD index indicated the closest overall post-match alignment in the U17 case (1.93), followed by U16 (1.98) and U15 (2.12); the U18 case showed the largest overall discrepancy (4.35). These values are descriptive and are not inferential agreement statistics.

Dimension-specific discrepancies varied by case. Particularly large positive discrepancies occurred for Support and Active Management in U18, while negative discrepancies for Negative Feedback and Passive Management indicated higher athlete rates than coach ratings in that case.

**Figure 2:**
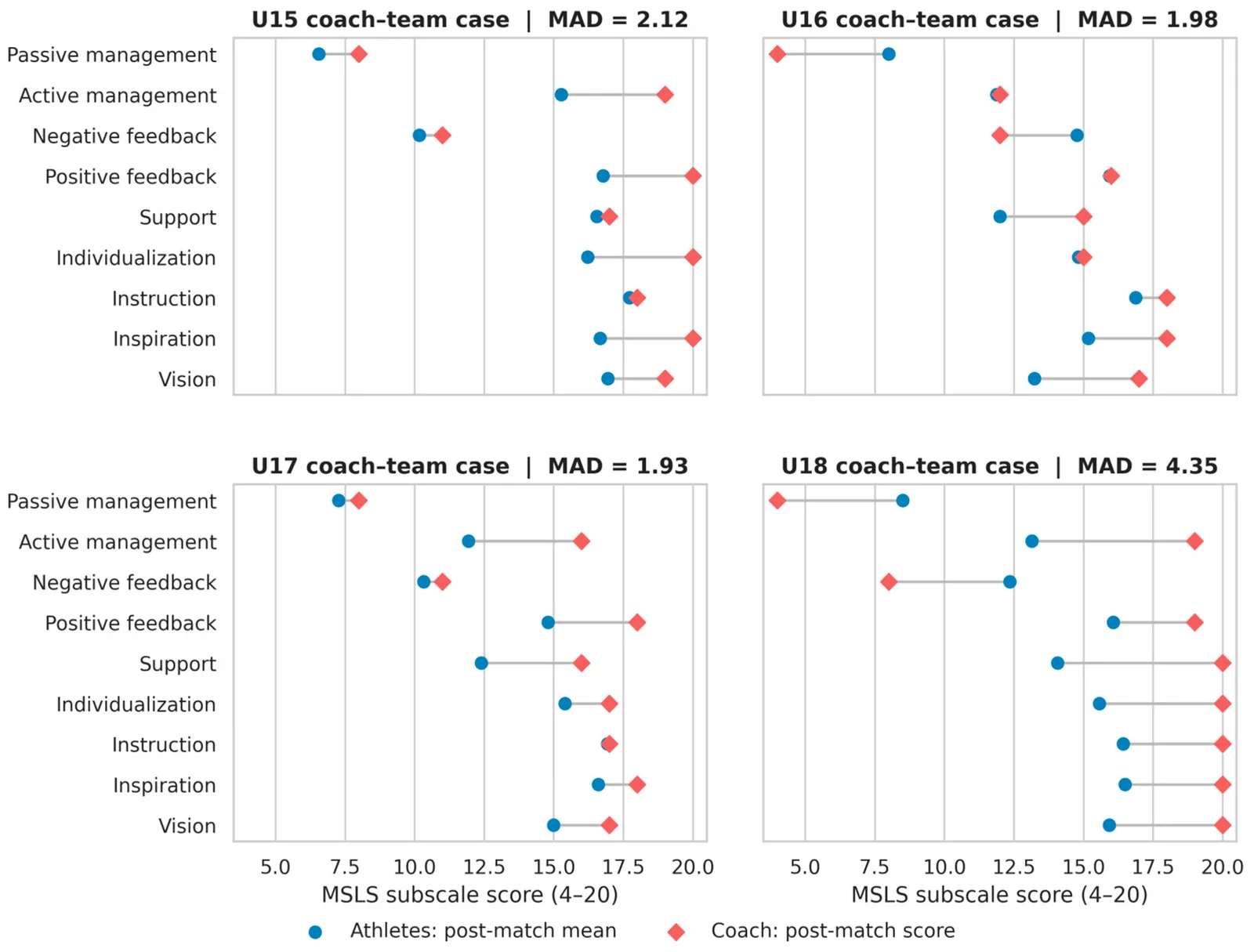
Case-specific post-match coach scores and corresponding athlete means across the nine MSLS dimensions. Horizontal distance between markers represents the descriptive discrepancy.

Figure 2 visualizes coach scores and corresponding post-match team means within each case.

### 3.5. Pre-Match and Post-Match Changes in Athletes’ Leadership Perceptions

Pooled pre–post comparisons are presented as exploratory individual-level analyses because athletes were clustered within four teams. Table 5, Panel A, reports medians, interquartile ranges, nominal *p*-values, Holm-adjusted *p*-values, and effect sizes, whereas Panel B provides case-wise descriptive medians.

After Holm adjustment, post-match scores were significantly lower for Inspiration (*p*Holm = 0.007), Individualization (*p*Holm = 0.005), and Support (*p*Holm = 0.012). Nominal differences in Vision, Instruction, and Positive Feedback did not remain statistically significant after adjustment.

Effect sizes ranged from negligible to moderate. The adjusted findings represent assessment-time differences in the pooled sample and cannot be attributed to match outcome, athlete satisfaction, playing time, performance, or affective state.

**Figure 3:**
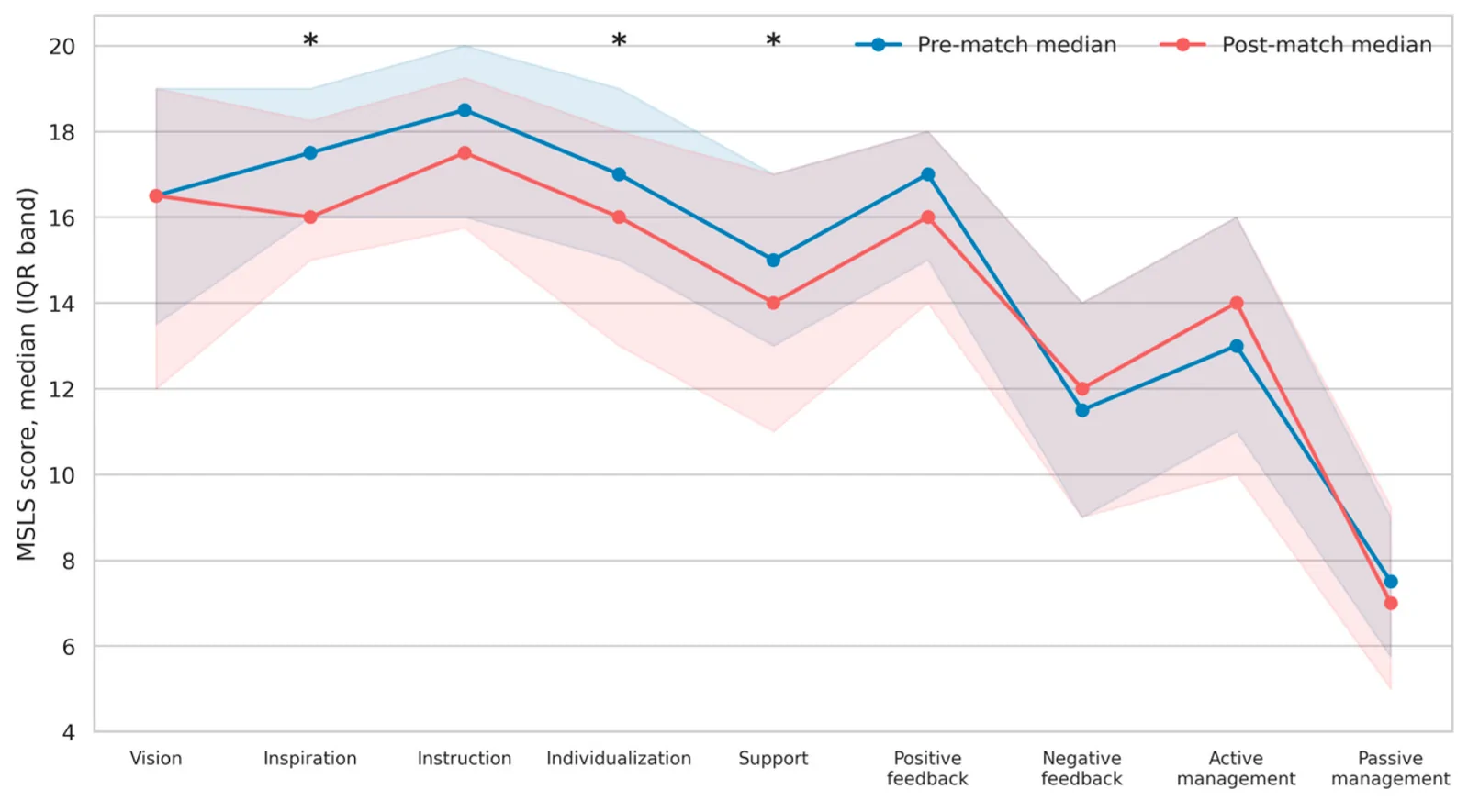
Pooled pre- and post-match MSLS medians with interquartile-range bands. Asterisks indicate comparisons that remained significant after Holm correction: Inspiration, Individualization, and Support.

The largest pooled effects were observed for Individualization (r = 0.433), Inspiration (r = 0.414), Support (r = 0.393), and Vision (r = 0.317); only the first three remained statistically supported after Holm correction. The pooled pre- and post-match median scores and their corresponding interquartile ranges across the nine MSLS dimensions are presented in Figure 3.

### 3.6. Observed Coaching Behaviors During Official Matches

Table 6 summarizes the absolute counts and relative frequencies of behaviors observed during one selected match for each coach–team case. Instruction was the most frequent category in each match (25.2–41.6%). These observations describe the selected matches only.

Other categories varied across cases, including Hustle, Praise, Scold, and Silence. Such variation may reflect match-specific circumstances and cannot be interpreted as stable behavioral profiles or differences attributable to age category.

Observed frequency indicates how often a coded action occurred; it does not establish the quality, appropriateness, or effectiveness of that behavior.

**Figure 4:**
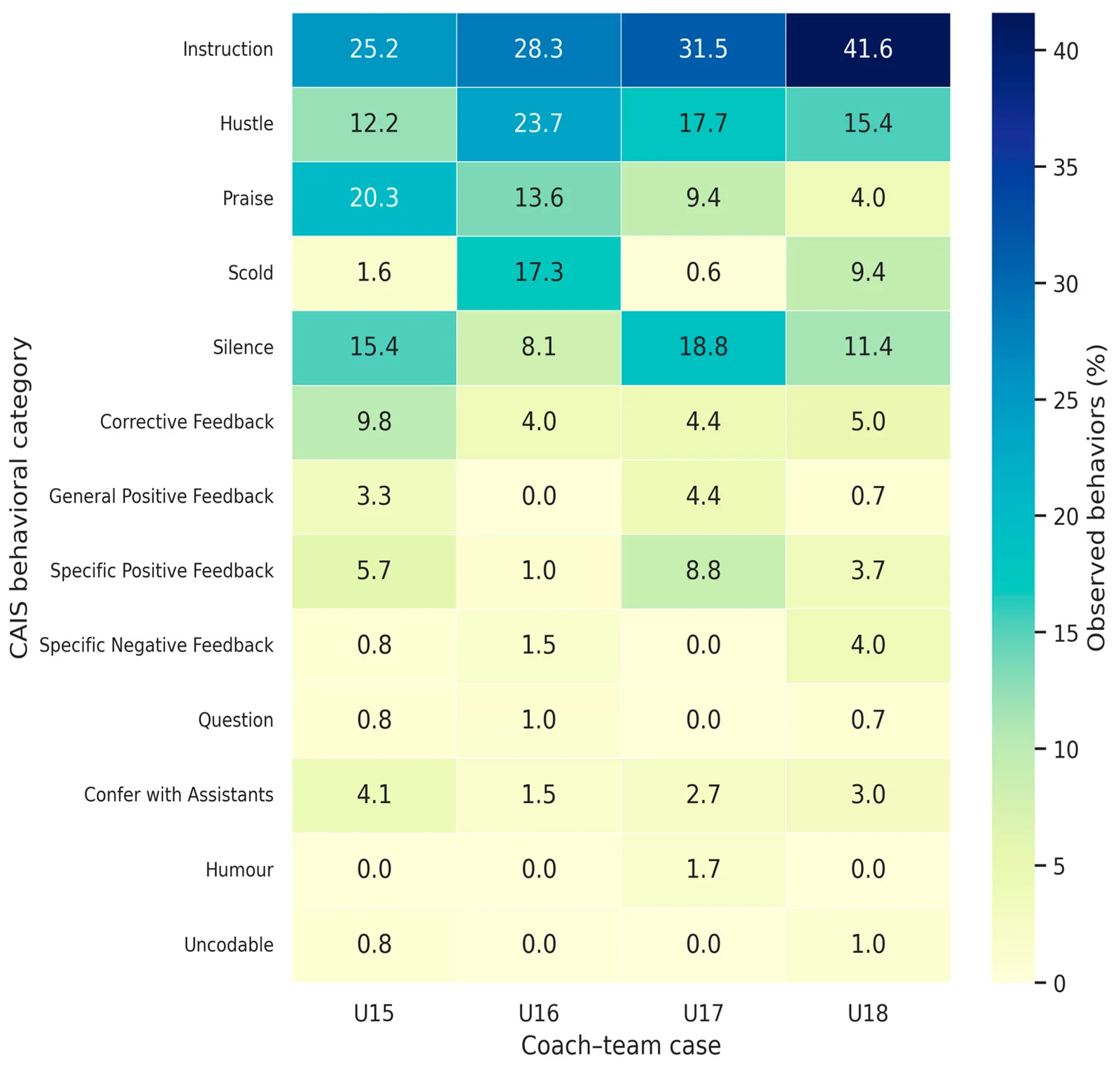
Heatmap of CAIS behavioral frequencies observed during one selected official match per coach–team case. Cell values are percentages of all behaviors coded within that match; darker color indicates a higher percentage. Frequencies should not be interpreted as habitual coaching behavior or behavioral quality.

Figure 4 presents the complete CAIS percentage distribution as a heatmap; darker cells indicate higher within-case percentages.

### 3.7. Exploratory Integration of Perceived and Observed Leadership

MSLS and CAIS findings were integrated through the predefined conceptual matrix in Table 7. The purpose was to describe parallel, partially parallel, contrasting, or non-comparable patterns without assuming construct equivalence.

Instructions showed a parallel descriptive pattern: it was rated highly in the MSLS and was the most frequent CAIS category in all four selected matches. This pattern does not constitute formal agreement and does not indicate instructional quality or effectiveness.

Positive Feedback showed only a partially parallel pattern because broad MSLS ratings were high while the frequencies of Praise and general/specific positive feedback varied by match. Negative Feedback also varied across cases.

Support, Active Management, and Passive Management had no direct CAIS equivalents. Silence was not treated as evidence of passive leadership, and corrective feedback was not treated as inherently negative.

Accordingly, cross-method integration provides complementary contextual description rather than validation, correlation, or agreement between the instruments.

## 4. Discussion

### 4.1. Principal Findings

This descriptive multi-case study examined coach–athlete perceptual alignment within four cases and complemented these data with preliminary observations from one selected match per coach. The revised analysis emphasizes case-specific discrepancies and cautious cross-method comparison rather than age-category effects or formal agreement.

The post-match MAD index was lowest in U17 and highest in U18, demonstrating heterogeneity among the four cases. After Holm correction, only Inspiration, Individualization, and Support retained evidence of lower post-match scores. Instruction was frequently observed in each selected match, but this frequency does not establish a stable coaching characteristic or instructional effectiveness.

The MSLS and CAIS findings offered complementary perspectives. Instruction produced a parallel descriptive pattern, whereas feedback-related findings were less consistent and several MSLS dimensions lacked direct CAIS equivalents. These patterns should not be interpreted as measurement convergence.

All findings are bounded by the four-case, single-academy design. They characterize the coach–team contexts investigated and cannot distinguish coach-, team-, match-, or developmental influences.

### 4.2. Leadership Perceptions and Coach–Athlete Alignment

Instruction was rated highly by athletes and coaches and was the most frequently coded behavior in each selected match. This constitutes a parallel descriptive pattern across complementary sources, not formal agreement. Although instructional behaviors are frequently reported in observational studies of soccer coaching, their frequency alone cannot establish instructional quality or effectiveness (Côté & Gilbert, 2009; C. Cushion et al., 2012; C. J. Cushion et al., 2012; Ford et al., 2010).

Athletes also reported comparatively favorable Vision, Inspiration, Support, and Positive Feedback scores. These findings describe perceived leadership within the four cases and should not be interpreted as evidence of broader coaching effectiveness (Gomes, 2014; Gomes & Resende, 2014; Gomes et al., 2021).

Case-specific discrepancies showed that coaches often rate selected supportive or management dimensions more favorably than their athletes. The pattern was not uniform across all dimensions or cases, and neither perspective should be treated as an objective criterion of accuracy (Alfermann et al., 2005; Bortoli et al., 1995; Gardner et al., 1996).

Support and feedback may be expressed across training sessions and longer-term relationships, whereas CAIS captured discrete actions during one match. This difference in temporal and conceptual scope helps explain why direct cross-method equivalence cannot be assumed (C. J. Cushion et al., 2012; Gomes et al., 2021).

The case-level discrepancy approach therefore provides a transparent description of perceptual distance, while the observational component supplies limited contextual information about the selected matches.

### 4.3. Assessment Timing and Complementary Measurement Approaches

After Holm correction, post-match scores remained significantly lower for Inspiration, Individualization, and Support. Vision, Instruction, and Positive Feedback reached nominal but not adjusted statistical significance and were therefore not interpreted as statistically significant findings.

The factors underlying these assessment-time differences cannot be determined from the available data. Although all four matches ended in home victories and no unusual events were reported, score progression, opponent strength, playing time, individual performance, satisfaction, and emotional state were unavailable. The observed differences may therefore reflect match-specific responses or other unmeasured influences.

The CAIS findings describe only the coaches’ behaviors during the selected matches. They do not establish habitual behavioral patterns and cannot be causally linked to changes in the pre- and post-match questionnaire scores.

The integration matrix clarifies the conceptual boundaries between the two instruments. Conceptual overlap was most direct for Instruction; feedback dimensions showed only partial correspondence, while Support and management dimensions had no direct CAIS counterparts.

Within individual cases, combining athlete feedback with match observation may support structured reflective practice, consistent with approaches that emphasize reflection as an important component of coach learning and professional development (Knowles et al., 2001), provided that neither source is treated as a formal assessment of coaching quality.

### 4.4. Practical Implications

The findings support cautious use of athlete feedback and match observation as complementary reflective tools. The frequent occurrence of Instruction in the selected matches should prompt examination of how and when instruction is delivered, not an assumption that frequent instruction is inherently effective.

Case-specific discrepancies may help coaches identify dimensions in which their intended leadership differs from athletes’ immediate post-match perceptions. Such feedback should remain contextual and should not be used as a standalone evaluation of coaching quality.

For coach education, repeated observations across matches and training sessions, combined with reliable multi-observer coding, would be more informative than a single recorded match. Video-based observation and feedback may be particularly useful in this process because they allow coaches to revisit specific interactions, compare intended and observed behaviors, and engage in evidence-informed reflection on their practice (Harvey et al., 2010; Wadsworth et al., 2020).

The present approach is therefore best viewed as a preliminary framework for structured reflection rather than a basis for normative or age-related conclusions.

### 4.5. Limitations and Future Directions

The study has several important limitations. First, the core observational sample comprised only four coach–team cases from one academy, with one coach representing each age category. The findings cannot distinguish age, coach, team, academy, or match effects and are not broadly generalizable.

Second, only one official match was observed per coach. Coaching behavior may vary with opponent strength, score progression, match importance, performance, substitutions, injuries, disciplinary events, and emotional intensity. Although all selected matches were home victories without reported unusual events, exact scorelines and several contextual indicators were unavailable.

Third, CAIS coding was completed by a single observer, without inter-rater or intra-rater reliability assessment. Coding consistency cannot therefore be independently verified, and the observational results must be treated as preliminary. The observer was blinded to MSLS findings during coding, but repeated independent coding would have strengthened reliability.

Fourth, athletes were clustered within four teams and shared the same coach and match context. The pooled Wilcoxon tests do not model this dependence and are interpreted only as exploratory individual-level comparisons. Holm correction reduced the risk of false-positive findings across the nine tests.

Fifth, the Romanian-language MSLS administration lacked documented formal validation or translation/back-translation. Internal consistency alone does not establish linguistic, cultural, or construct validity; therefore, all MSLS-based findings should be interpreted cautiously. Finally, MSLS and CAIS are non-equivalent in construct and temporal scope; their integration is descriptive and cannot establish formal agreement. Future research should include larger multi-academy samples, multiple coaches per category, repeated matches and training sessions, validated language procedures, and independent observers with reported agreement coefficients.

## 5. Conclusions

Within four coach–team cases, post-match coach–athlete perceptual alignment varied across leadership dimensions and cases. The U17 case showed the smallest overall MAD index and U18 the largest. After Holm correction, post-match ratings were lower for Inspiration, Individualization, and Support.

Instruction was frequently observed in each selected match and was rated highly in the MSLS, representing a parallel descriptive pattern across non-equivalent measurement approaches. This finding does not demonstrate formal agreement, habitual coaching behavior, instructional quality, or effectiveness.

The results are preliminary and specific to four cases. Repeated multi-observer observations and larger case samples are required before broader conclusions regarding youth soccer coaching leadership can be drawn.

## Figures and Tables

**Table 1:** Demographic and competitive characteristics of participants.

Characteristic	U15	U16	U17	U18	Total
Number of players	18	17	15	14	64
Number of coaches	1	1	1	1	4
Time working with current team	≈2 years	≈2 years	≈2 years	≈2 years	
UEFA A licence	1	1	0	1	3
UEFA B licence	0	0	1	0	1
Observed match venue	Home	Home	Home	Home	
Observed match result	Win	Win	Win	Win	
Opponent	Same category	Same category	Same category	Same category	
Reported unusual events	None	None	None	None	

**Table 2:** Internal consistency estimates for the Multidimensional Scale of Leadership in Sport (MSLS) subscales using Cronbach’s alpha (α) and McDonald’s omega (ω).

MSLS Dimension	Items (n)	Cronbach’s α	McDonald’s ω
Vision	4	0.926	0.926
Inspiration	4	0.801	0.809
Instruction	4	0.874	0.875
Individualization	4	0.848	0.853
Support	4	0.872	0.875
Positive Feedback	4	0.778	0.790
Negative Feedback	4	0.824	0.823
Active Management	4	0.852	0.853
Passive Management	4	0.649	0.689

**Table 3:** Combined pre- and post-match athlete MSLS scores across the four coach–team cases (mean ± standard deviation).

Leadership Dimension	U15	U16	U17	U18
Vision	17.42 ± 2.72	13.53 ± 4.21	15.60 ± 3.87	15.89 ± 3.80
Inspiration	17.25 ± 2.47	15.71 ± 2.91	17.03 ± 2.51	16.75 ± 3.11
Instruction	18.22 ± 1.81	16.85 ± 3.14	17.23 ± 2.34	16.54 ± 3.66
Individualization	16.67 ± 2.90	15.21 ± 3.42	15.97 ± 3.56	15.93 ± 3.74
Support	16.89 ± 2.95	12.26 ± 3.95	13.27 ± 4.90	14.43 ± 2.85
Positive feedback	16.97 ± 2.18	16.24 ± 2.54	15.33 ± 3.89	16.32 ± 2.79
Negative feedback	9.89 ± 2.27	14.35 ± 3.64	10.40 ± 3.52	12.14 ± 2.26
Active management	15.44 ± 2.70	11.82 ± 3.66	11.87 ± 4.27	13.21 ± 3.79
Passive management	6.61 ± 2.35	8.09 ± 2.82	7.23 ± 2.52	8.04 ± 2.65

Values combine both athlete assessment time points and are presented as mean ± standard deviation.

**Table 4:** Post-match case-specific coach–athlete perceptual alignment. Discrepancy = coach score − corresponding team mean; values closer to zero indicate closer descriptive alignment. MAD = mean absolute discrepancy across nine dimensions.

**MSLS Dimension**	**Coach Score**	**Athlete Post-Match Mean**	**Discrepancy**	**MSLS Dimension**	**Coach Score**	**Athlete Post-Match Mean**	**Discrepancy**
**Panel A. U15 Coach–Team Case**				**Panel B. U16 Coach–Team Case**			
Vision	19	16.94	+2.06	Vision	17	13.24	+3.76
Inspiration	20	16.67	+3.33	Inspiration	18	15.18	+2.82
Instruction	18	17.72	+0.28	Instruction	18	16.88	+1.12
Individualization	20	16.22	+3.78	Individualization	15	14.82	+0.18
Support	17	16.56	+0.44	Support	15	12.00	+3.00
Positive feedback	20	16.78	+3.22	Positive feedback	16	15.94	+0.06
Negative feedback	11	10.17	+0.83	Negative feedback	12	14.76	−2.76
Active management	19	15.28	+3.72	Active management	12	11.88	+0.12
Passive management	8	6.56	+1.44	Passive management	4	8.00	−4.00
Mean absolute discrepancy (MAD)			2.12	Mean absolute discrepancy (MAD)			1.98
**Panel C. U17 Coach–Team Case**				**Panel D. U18 Coach–Team Case**			
Vision	17	15.00	+2.00	Vision	20	15.93	+4.07
Inspiration	18	16.60	+1.40	Inspiration	20	16.50	+3.50
Instruction	17	16.93	+0.07	Instruction	20	16.43	+3.57
Individualization	17	15.40	+1.60	Individualization	20	15.57	+4.43
Support	16	12.40	+3.60	Support	20	14.07	+5.93
Positive feedback	18	14.80	+3.20	Positive feedback	19	16.07	+2.93
Negative feedback	11	10.33	+0.67	Negative feedback	8	12.36	−4.36
Active management	16	11.93	+4.07	Active management	19	13.14	+5.86
Passive management	8	7.27	+0.73	Passive management	4	8.50	−4.50
Mean absolute discrepancy (MAD)			1.93	Mean absolute discrepancy (MAD)			4.35

**Table 5:** Pre- and post-match athlete MSLS scores. Panel A: pooled Wilcoxon signed-rank tests with Holm correction. Panel B: case-wise medians (pre/post), reported descriptively without case-level inference.

**Panel A. Pooled Pre–Post Comparison (N = 64)**						
**Dimension**	**Pre Median (IQR)**	**Post Median (IQR)**	**|z|**	***p***	***p*** **Holm**	**r**
Vision	16.5 (13.5–19)	16.5 (12–19)	2.534	0.011	0.068	0.317
Inspiration	17.5 (16–19)	16 (15–18.25)	3.312	<0.001	0.007	0.414
Instruction	18.5 (16–20)	17.5 (15.75–19.25)	2.191	0.028	0.142	0.274
Individualization	17 (15–19)	16 (13–18)	3.464	<0.001	0.005	0.433
Support	15 (13–17)	14 (11–17)	3.146	0.002	0.012	0.393
Positive feedback	17 (15–18)	16 (14–18)	2.051	0.040	0.161	0.256
Negative feedback	11.5 (9–14)	12 (9–14)	1.297	0.195	0.584	0.162
Active management	13 (11–16)	14 (10–16)	0.210	0.834	0.834	0.026
Passive management	7.5 (5.75–9)	7 (5–9.25)	0.827	0.408	0.816	0.103
**Panel B. Case-Wise Descriptive Medians (Pre/Post)**						
**Dimension**	**U15**	**U16**	**U17**		**U18**	
Vision	19/18	12/12	16/14		16.5/17.5	
Inspiration	18/17	16/16	18/17		17.5/16.5	
Instruction	19/18	17/17	18/17		17.5/16.5	
Individualization	17.5/16.5	16/15	18/16		17/16.5	
Support	18.5/17	13/12	15/12		15/14	
Positive feedback	17.5/17	17/16	17/15		17/16.5	
Negative feedback	9.5/10	15/16	10/12		11.5/13	
Active management	15/15.5	12/11	13/12		13/12	
Passive management	6/6	8/7	8/6		7.5/8	

**Table 6:** Relative frequencies (%) of CAIS-coded behaviors observed during one selected official match for each coach–team case.

Coaching Behavior	U15, n (%)	U16, n (%)	U17, n (%)	U18, n (%)
Instruction	31 (25.2)	56 (28.3)	57 (31.5)	124 (41.6)
Hustle	15 (12.2)	47 (23.7)	32 (17.7)	46 (15.4)
Praise	25 (20.3)	27 (13.6)	17 (9.4)	12 (4.0)
Scold	2 (1.6)	34 (17.2)	1 (0.6)	28 (9.4)
Silence	19 (15.4)	16 (8.1)	34 (18.8)	34 (11.4)
Corrective Feedback	12 (9.8)	8 (4.0)	8 (4.4)	15 (5.0)
General Positive Feedback	4 (3.3)	0 (0.0)	8 (4.4)	2 (0.7)
Specific Positive Feedback	7 (5.7)	2 (1.0)	16 (8.8)	11 (3.7)
Specific Negative Feedback	1 (0.8)	3 (1.5)	0 (0.0)	12 (4.0)
Question	1 (0.8)	2 (1.0)	0 (0.0)	2 (0.7)
Confer with Assistants	5 (4.1)	3 (1.5)	5 (2.8)	9 (3.0)
Humour	0 (0.0)	0 (0.0)	3 (1.7)	0 (0.0)
Uncodable	1 (0.8)	0 (0.0)	0 (0.0)	3 (1.0)
Total	123 (100.0)	198 (100.0)	181 (100.0)	298 (100.0)

Values are presented as absolute coded-event counts, with percentages of all behaviors coded within the corresponding match in parentheses. Total coded events were 123 for U15, 198 for U16, 181 for U17, and 298 for U18. Percentages may not total exactly 100% because of rounding.

**Table 7:** Exploratory MSLS-CAIS integration matrix showing conceptual links, descriptive patterns, and interpretive limitations.

MSLS Dimension	CAIS Category/Categories Considered	Conceptual Rationale	Descriptive Cross-Method Pattern	Interpretive Limitation
Instruction	Instruction	Direct verbal/tactical guidance	Parallel: high MSLS ratings; highest CAIS frequency in all cases	Frequency does not indicate quality or effectiveness.
Positive Feedback	Praise; general/specific positive feedback	Observable reinforcement	Partially parallel: high MSLS ratings; CAIS frequencies varied	Broad perception versus discrete match events.
Negative Feedback	Scold; specific negative feedback	Observable reprimanding responses	Variable across cases	Corrective feedback is not inherently negative.
Support	No direct equivalent	Relational support may extend beyond match events	Not directly comparable	Construct correspondence is indirect.
Active Management	Hustle; question; confer with assistants	Contextual match-management actions	No formal alignment classification	CAIS does not reproduce the MSLS construct.
Passive Management	Silence (context only)	No overt coded coaching action	No formal alignment classification	Silence cannot establish passive leadership.

## Data Availability

The data presented in this study are available from the corresponding author upon reasonable request. The data are not publicly available due to privacy and ethical restrictions involving minor participants and competitive team environments. Supplementary statistical materials, including raw datasets, Microsoft Excel files, and JASP analysis files supporting the findings of this study, are available from the corresponding author upon reasonable request.

## Abbreviations

The following abbreviations are used in this manuscript:

MSLS	Multidimensional Scale of Leadership in Sport
CAIS	Coach Analysis and Intervention System
SDT	Self-Determination Theory
MAD	Mean Absolute Discrepancy
U15	Under-15 age category
U16	Under-16 age category
U17	Under-17 age category
U18	Under-18 age category
JASP	Jeffreys’s Amazing Statistics Program
